# Working and learning from home during COVID-19: International experiences among social work educators and students

**DOI:** 10.1177/00208728211051412

**Published:** 2021-11-17

**Authors:** Michael Wallengren-Lynch, Lena Dominelli, Carin Cuadra

**Affiliations:** Malmö University, Sweden; University of Stirling, UK; Malmö University, Sweden

**Keywords:** coping strategies, COVID-19, social work educators, social work students, well-being, working and learning from home

## Abstract

This research seeks to explore the experiences of social work educators and students working and learning from home. The findings, from an international survey sample of 166 educators and students, showed that the respondents faced issues with private and personal boundaries, felt the impact of working and learning from home on both physical and emotional levels, and experienced challenges to what was expected of them. The respondents primarily used two types of coping mechanisms to manage these challenges. These findings contribute to a broader discussion of the impact of working and learning from home and are relevant for education administrators responsible for their employees’ and students’ well-being.

## Introduction

Educators and students of social work have had to adapt their work and life realities to accommodate the necessary restrictions imposed by governments across the world responding to COVID-19. This created significant changes in working and learning practices within social work education as working and learning from home has become the norm for the majority of social work educators and students globally. Working and learning from home has to a large degree, limited some types of interaction that educators and students can have, especially face-to-face ones. The data show a renaissance in the valuing of face-to-face relationships and innovations in education delivery that have been stifled through online environments. Also, the exacerbation of digital divides previously masked by person-to-person engagement makes it very challenging to use critical pedagogies including student participation and engagement and the involvement of service users in social work education processes. The experience of working and learning from home during COVID-19 has been influenced by a number of factors including home-schooling children, having limited physical exercise, sporadic communication with co-workers and inadequate workspaces at home (Xiao et al., 2021).

The aims of this research are to articulate and examine the experiences of working and learning from home during the COVID-19 pandemic among educators and students from international social work communities. Besides identifying these experiences, we consider how the respondents coped with the challenges associated with working and learning from home during this time.

## Implications for working and learning under the conditions of the COVID-19 pandemic

COVID-19 has brought levels of uncertainty that is unfamiliar to many people. Afrouz (2021) argues that we need to find ways to introduce the experience of uncertainty into social work teaching. He argues that it is essential that we help students prepare for a world where uncertainty regarding many issues, such as climate change and global pandemics, is a recurring theme. Many social work students have felt the impact of this uncertainty and feel like their anchors in life have gone (Cole et al., 2021). We know that social students normally take time to adjust to university demands so we can anticipate that COVID-19 has made the transition even more challenging (Stanley and Bhuvaneswari, 2016; Vungkhanching et al., 2016). As social work educators, we are forced to think creatively and adapt technology to meet our pedagogical goals (Kourgiantakis and Lee, 2020). The adaption is also echoed by Mclaughlin et al. (2020), who state that COVID-19 ‘sparked opportunities for innovation, creativity, and humanistic endeavours in meetings the needs of the students and moving forward in delivering social work education remotely and virtually’ (p. 975). The impact this has had on social work students has yet to be fully understood. However, early research shows that online teaching identified increasing inequality, and the differences in student learning styles have become more visible (De Jonge et al., 2020). In order to be adaptive and survive, social work educators and students have retreated to their homes to continue teaching, researching and learning. The kitchen table has turned into an office desk or classroom, bringing new work and study realities for many.

Working and learning from home has been welcomed by some and experienced as stressful by others. Early studies on the impact of remote working on social work professionals in the United Kingdom show that working from home has blurred the lines between private and professional spaces (https://www.theguardian.com/society/2020/sep/22/nine-at-night-laptop-still-open-social-work-pandemic). An international survey conducted by the International Federation of Social Workers (IFSW) found that ‘social workers have struggled to continue to do their work – having to adapt and innovate to meet new needs and reprioritise the most urgent and important aspects of their traditional roles’ (Banks et al., 2020: 570). Other international social work organisations have produced reports on the day-to-day life of social workers, educators and students during this pandemic, for example, the compilation of in-country reports available on the International Association of Schools of Social Work (IASSW) website, https://www.iassw-aiets.org/covid-19/. Burnout within the profession has reached even higher levels of risk during the pandemic, and developing coping tools is considered essential (Peinado and Anderson, 2020).

There is a distinction between working from home and learning from home and yet the two realities, especially during COVID-19, have a lot in common. For that reason, and due to the lack of research regarding students’ views of learning from home, we focus on the ‘working from home’ literature to help us illuminate the experiences of both teachers and students. Working from home is not a new concept (Vyas and Butakhieo, 2020). With the recent improvements in technology, an increasing number of professionals have begun to work from home on a regular basis. Some studies point out that ‘telework can reduce turnover rates and increase employees’ productivity, job engagement, and job performance’ (Vyas and Butakhieo, 2020: 6). A recent study by Dingel and Neiman (2020) revealed that 37 percent of jobs in the United States could be completed at home during the COVID-19 pandemic, including financial work, business management, and professional and scientific services. However, this ignores the realities facing essential workers who were precluded from working from home by the very nature of their job, for example, professionals providing health and social care services, porters, cleaners, cooks in essential services, drivers in the haulage and transportation industries, and workers in the food and pharmaceutical sectors.

Working from home pre-COVID-19 and during COVID-19 had some differences. In situations where employers allowed it, working from home pre-COVID-19 became an individual choice, for example, in academia unless classes or meetings were scheduled at that time. Nonetheless, with the onset of the COVID-19 crisis, a wider range of tasks, including all classroom sessions and meetings, were undertaken remotely. Consequently, an increasing amount of research has focused on the experiences of working and learning from home under its special conditions. The current working climate promotes few social contacts, working in isolation and loneliness. There is a gap in the existing literature regarding social work educators’ and students’ experiences during COVID-19 that this article attempts to fill. Other professionals are covered in the literature, for example, those located in business. Moreover, the experiences of diverse sectors of the population differ. Xiao et al.’s (2021) research in the United States indicated that overall, there was ‘decreased physical and mental wellbeing status and an increased number of physical and mental health issues following the transition to working from home during the first four months of COVID-19’ (p. 189). Furthermore, from the sample of 988, Xiao et al. (2021) saw that ‘reduced physical wellbeing was moderately correlated with reduced mental wellbeing’ (p. 189). These outcomes were directly impacted by gender and income levels. These authors reported that having two or more new physical and mental issues occurred more often among women than men workers generally and/or workers with higher income levels. Their findings correlated with another recent survey by Messenger et al. (2017) which noted that women ‘workers have a higher risk of depression while working from home during a pandemic’ (p. 12). Moreover, Xiao et al. (2021) concluded that working from home may be more challenging for women since they tend to be more responsible for household chores and other home activities. Working mothers can feel double pressures when working from home due to lack of support with home-schooling and children’s care alongside their responsibilities for daily maintenance of the family such as cooking and cleaning. Women still carry the burden of household chores that support individual and family well-being even while working from home.

Relationships among colleagues at work are also important concerns covered in the literature. In a pre-pandemic study, Collins and Moschler (2009) found that workers who were isolated from their co-workers felt its impact alongside managerial concerns about reductions in productivity among those working from home. Moreover, a study by Gajendran and Harrison (2007) showed that relationships among co-workers could be harmed by the isolation engendered by working from home. Other significant findings of significant relevance to today’s situation focus on employee distraction caused by having young children or family members around while working at home (Baruch, 2000; Kazekami, 2020). This, along with the blurred boundaries between work and family life, can lead to overwork, especially among women.

In a similar vein, the management of boundaries between work and family of remote workers studied by Eddleston and Mulki (2017) revealed that working from home reduces the ability of remote workers to disengage from work. These findings were supported by Vyas and Butakhieo (2020) who found that ‘the drawbacks of working from home, include the blurred line between work and family, distractions, social isolation, employees bearing the costs related to working from home’ (p. 8). This includes increased costs for energy consumption, although for some, it may be accompanied by reduced transportation and food costs. Oakman et al.’s (2020) findings from a rapid systemic review ‘suggest that the impact of working from home on individuals’ mental and physical health vary considerably’ (p. 1825). Oakman et al.’s (2020) conclusions focused on the implications for practice related to ‘organisational support, in particular the need for management training for the supervision of employees working from home’ (p. 1825). In addition, this review pointed out that co-worker support is essential, both at formal and informal levels. Further support is needed with technological issues, boundary management and in formalising expectations. This is additional to the support that social workers normally require and receive from both peers and line managers to manage their workloads and provide services.

Myers et al. (2020) examining risk regarding stress and self-care among social work educators argue that ‘students, academics, and human service workers are at risk of burnout, compassion fatigue, secondary traumatic stress, and vicarious traumatisation due to the nature and magnitude of their workload’ (p. 2). While social work faculty and staff may not be experiencing the stress of being in the human services workplace, they navigate similar risks for burnout and fatigue (Miller et al., 2018). This ties in with McFadden et al.’s (2021) observations that

it quickly became evident that the negative impact of COVID-19 was felt not just at the organisational level, but also at the individual level, as studies of the mental health and wellbeing of health and social care staff started emerging. (p. 2)

And, social work communities generally had been experiencing high levels of work-related stress before the outbreak of COVID-19, and this was reflected in high rates of turnover among employees and high vacancy levels among diverse agencies (Frieiro Padín et al., 2021).

The recommendations provided by these studies are generally similar to those summarised in the reflections of Xiao et al. (2021). The research literature suggests that as people under COVID-19 are restricted from many activities, it is important to continue moderate exercise while working from home, including walking, taking active short breaks and playing with their children. All of these activities can be beneficial for health and well-being.

## ‘In this together’?

Whether people are ‘in the same boat’ as those around them can affect their experience of working and learning from home, and, despite difficulties encountered, can provide opportunities for growth (Arnold et al., 2005). However, for many working from home during COVID-19, these opportunities have been few. Working from home is assessed largely in terms of organisational considerations such as IT support, connection to colleagues, relationships with management and individual/family factors including household characteristics such as the size of the living area, number of family members sharing the same accommodation, sharing computers and/or phones, and the number and age of children in the household (Vyas and Butakhieo, 2020).

This section seeks to understand respondents’ experiences and explain key concepts related to self-care and coping during their dramatic change of work and study circumstances. Personal self-care encompasses practices for health and personal well-being that may involve engaging in a fulfilling hobby, spending time with a supportive friend, exercising, using humour and meditating. Professional self-care is the process of purposefully engaging with practices that promote an effective professional self, giving attention to workloads, professional development, time-management and resource availability. Folkman (1984) sees ‘coping as referring to cognitive and behavioural efforts to master [*sic*], reduce, or tolerate the internal and/or external demands that are created by the stressful transaction’ (p. 843). An important feature of this definition is that coping is defined independently of its outcome or structural constraints, thus emphasising individual resilience or the capacity to cope. Coping refers to efforts to manage demands, regardless of the success of those efforts. Coping is viewed in this formulation as having two major functions: regulating emotions or distress (emotion-focused coping) and the management of the problem that is causing the distress (problem-focused coping). For McFadden et al. (2021), the coping process consists of two parts: the primary appraisal of the event as being harmful or threatening; and the secondary appraisal of one’s own coping options or mechanisms that can be used to deal with the potentially stressful event or situation. Mette et al. (2020) echo Folkman when describing coping as

cognitive and behavioural efforts made to master [*sic*], tolerate or reduce external and internal demands, as well as conflicts among them. Coping strategies either aim to manage the stress-inducing problem (problem-focused) or regulate emotions or distress caused by the problem (emotion-focused). (p. 3)

Problem-oriented strategies refers to employees’ work tasks and content, social relations, and personal strategies which seeks instrumental support from colleagues and superiors to deal with stressful situations and concrete problems (Mette et al., 2020: 3). Emotionally orientated activities include engagement in leisure pursuits, social relations, acceptance, ruminating and seeking informal social support from family and friends, to alleviate negative emotions (Mette et al., 2020: 4). Several emotion-oriented coping strategies used by the workers in the Mette et al.’s (2020) study involved cognitive components, such as gaining mental distance from work, accepting unchangeable situations and focusing on positive experiences.

People use a variety of coping strategies at any given time based on a complex variety of factors. The unique situation of COVID-19 presents a new set of factors that impact working and learning from home. MacIntyre et al.’s (2020) survey of 600 language teachers regarding their stress factors and coping mechanisms to teaching online during COVID-19 found that coping was considered a healthy way of managing stress. Taylor et al. (2020) explain avoidant coping strategies as a person’s efforts to avoid a particular stressor, deemed as unfavourable, by using strategies that have an overall negative effect. Applying such strategies as a way of coping with working and learning from home during COVID-19 raises the possibility of potential costs attached to using avoidant coping strategies.

This section has examined what is known about working from home and its impact on well-being. We explored the coping mechanisms that were utilised and also identified two ways that individuals used to cope with stress factors. The literature referred to is relatable to the experiences that the respondents in this research described, and to the two forms of coping that can be used to frame the strategies they drew upon to deal with working from home during this pandemic.

## Method

The answer to the research questions outlined in this article draws on data gathered from a survey questionnaire disseminated through the IASSW website. The IASSW is an international association of tertiary-level social work educational programmes called ‘schools’ and social work educators. Together, these schools form its social work educational community. The researchers sought to hear as many voices from this community as possible. The survey had over 40 questions, in the English language, with the majority as open-ended questions to invite participants to write freely and at-length. The survey, using the software SUNET, was opened in mid-December and closed in mid-January. During that time, 166 people, representing 32 countries (see Table 1) from across all continents, replied providing a snapshot of respondent’s experiences during that time. The vast majority of those who replied (80%) were women and evenly spread across the age range of 25–61 years. Close to half of the sample had doctorate degrees in social work or social work–related subjects. Over half of the respondents were either full-time or part-time educators. Another 15 percent of respondents considered themselves a combination of educator and practitioner. Overall, the sample had a higher representation of educators than students and practitioners, with 22 percent of respondents being practitioners, working part-time or full-time, and 14 percent full-time students.

**Table 1.** List of countries who participated in the research.

Israel	Sweden	India	United States of America	Canada	Botswana
Australia	Philippines	Guyana	Portugal	Belgium	India
Greece	Slovenia	Japan	Chile	Malaysia	Germany
Norway	Sri Lanka	Malawi	Nigeria	United Kingdom	Indonesia
Zambia	Scotland	Wales	Kenya	Finland	Russia
Romania	South Africa				

The data, in the form of respondent’s statements, were imported to NVivo, and subsequently themes were created using an inductive approach, as suggested by Braun and Clarke (2006). Codes were identified first and subsequently used to create the themes.

The respondents are not identifiable nor known to the researchers, and since only those that wished to respond did, providing whatever information they were willing to share, separate ethical approval was not required. However, ethical consideration was given to confidentiality. Each respondent was presented with a consent form and information about the research before moving to the online survey. The respondent identities were unknown to the researchers. The researchers were conscious of the moral considerations regarding asking people to participate in the research study in the middle of a pandemic. However, given the research methodology and self-selecting sample strategy, the survey was non-invasive and did not compromise moral or ethical positions. In other words, the respondents were not ‘purposively recruited’ to the study; instead, they choose to volunteer their time to answer the survey. We considered it necessary not to burden potential respondents, given the challenges many social work educators and students had been experiencing during the COVID-19 pandemic. This methodological approach proved to be sufficient for our research needs.

Those who replied were self-selecting and presumably are weighted towards those who felt they had something to say. This raises the issue of who were those who chose not to reply, and what are the implications of this for our findings? However, because it is difficult for us to ascertain the response to this point, we will not speculate upon it, but highlight it as an issue for future research.

The descriptive statistics were generated automatically by the survey system used to administer the survey.

## Analysis and discussion

In this section, we will briefly present a descriptive analysis of the findings. From this, we will consider the themes generated by the data and finally show how the respondents’ use of coping strategies could be categorised into two types: problem-oriented and emotionally-oriented.

The results from the study showed that over 55 percent of those surveyed considered that their stress levels were much higher now than before the pandemic. Over 55 percent had issues with sleeping now compared to the period before the pandemic. Their responses ranged from ‘some levels of difficulty’ (52%) of sleeping to ‘significant issues’ (22%) in sleeping. Over 53 percent of respondents had either a family member, colleague or close friend who had caught COVID-19. Over 13 percent of the respondents had lost a close friend or family member to COVID-19. Over 50 percent felt that increased time spent working from home had negatively impacted their home life.

On the educational front, over 83 percent of respondents had challenges in finding placements for their students as many agencies were not accepting any. Many creative solutions were required to ensure that students graduated on time and sadly, in some instances, student graduations were postponed.

## Managing boundaries between home and work

The work and study reality has changed for many respondents. The spheres of the private and the public have merged, mediated by online classrooms and Zoom meetings. For some educators, this meant working longer hours, the expectation of being always accessible, including for those in an environment of poor Internet connectivity. Others faced a substantial rise in workloads. For these, the move online meant that the ‘workload at the university has literally increased 40% and it is extremely difficult to keep up’. The boundaries between home and work life were forcibly merged for many educators. Some of the following quotes from respondents exemplify the challenges they experienced: ‘there is no boundary between work and home and being locked down for so long made it particularly hard to separate out these domains of life’. Another respondent commented ‘there is no longer a separation of any kind between work and personal life. I have rearranged my house to allow for a home office. I have work stuff all over my house’. For others, it ‘felt like work requisitioned our homes; much harder to switch off and separate work from private life’. The unplanned reorganisation of one’s private space with work furniture and equipment provides clear images of the infiltration of the work sphere into the private domain. The freedom to choose this work arrangement has been removed from respondents.

The shift from a pre-COVID-19 world has, for many, been a dramatic time. In one respondent’s words, it has had a ‘total impact – from going to university every day and seeing colleagues and students to doing everything online has been a tremendous shift’. For many respondents, even after acknowledging the sudden transition to online life, many continued to struggle with practical questions, particularly those pertaining to the boundaries around physical space. This is explained by the following quote from one of the respondents.

It is extremely difficult to balance the different spaces in our house, as none of us have an office or desk – so we were juggling [at] the kitchen table or just sitting on our beds studying all day. It was also really difficult to manage differential levels of concern and needs around COVID safety. It became unmanageable and I have since moved into a one-bedroom apartment. It’s much better in terms of being able to study, but I also feel quite isolated and lonely now and it is also significantly more expensive so I am quite financially precarious, having to use our own equipment to teach and research and practice from – [the] financial burden of buying equipment/internet use, finding space that was appropriate to teach from – for those of us with children, it was nearly impossible.

Working like this, where many ‘felt trapped at home’ as one respondent put it, has an impact on family members. For instance, ‘the amount of time that my family have to remain quiet when I am teaching . . . so the impact on them is greater’, and on a personal level ‘my home is now a stressful place, it is no longer my haven of joy and peace’ and even with clients ‘I do not have space to work from home and no privacy to do my sessions with clients or my teaching from home’. The significance that the respondents have given to the idea of boundaries is important. Boundaries enabled the respondents to have control of the different spheres in their lives. Losing this sense of control (e.g. Xiao et al., 2021) can have implications on one’s mental well-being. This has knock-on effects on other aspects of one’s life, some of which we will explore in the next theme.

## Impact on the physical and emotional self

The experience of working from home for social work educators and students was felt on multiple levels. From a physical point of view, given the confinement of many to their homes and the increased time spent in front of a computer, the process has impacted them in worrying ways. For instance, many respondents commented on physiological reactions such as heart ‘racing’, concentration problems, exacerbation of pre-existing conditions including sciatica, getting less physical exercise, ‘sleep pattern [that] is totally disrupted, not falling asleep until [the] wee hours of the morning often, need for sleeping pills’, ‘more fatigue physically & [*sic*] emotionally more malaise’. This impact on the physical body is documented in the research cited earlier such as Gajendran and Harrison (2007), where it was also pointed out that the impact of working from home can vary and is dependent on many factors including pre-existing conditions such as stress. This is an important factor to consider when trying to understand the impact that working from home during COVID-19 has had on educators and students.

On an emotional level, the respondents commented that they ‘fe[lt] more disconnected and overwhelmed, less engaged, supported, motivated, or inspired’. Life has changed dramatically for some and impacted the quality of their everyday life. One respondent expressed this sentiment as, ‘life has less texture, no hugs’. For some, working from home feels like ‘prison where one is working 24/7’. This has led to feelings of depression and of being overwhelmed, leading some to feel the ‘inability to take care of all tasks that should be taken care of’. For over 50 percent of the respondents, a close family member had contracted COVID-19. Combining the worry connected to this with work stress, respondents reported increased levels of anxiety.

Some respondents were able to see that the pandemic has made them appreciate their loved ones more and that the pandemic has enabled them to have also grown as individuals. One respondent commented that, ‘I have also learnt so much about myself while working from home’. Generally speaking, however, most of the responses referred to ‘sense of loneliness, lack of motivation and of doing more than before COVID’.

The impact of working from home is also felt on an interpersonal level with family and work colleagues. One respondent commented that

I am increasingly irritable and short at home, notice more arguments with family and a very low tolerance for anything ‘extra’. I take out the stress I am experiencing on anyone, so I tend to isolate as much as possible.

Another commented that, ‘my partner sees me in work mode and that kills any romance’. From a work perspective, it was also apparent that, ‘it has been extremely difficult for me to build relationships with my team’.

But there were also examples in which respondents recognised that working from home has actually helped their interpersonal relationships with family. One commented that ‘COVID-19 had a positive effect on my home life in some ways: ‘my college-age daughter came back to live at home for 6 months and it was nice to have her there’. Another commented that

We’re all at home now. That’s been nice in some ways. I feel closer to my teenage son. But on the other side, we have been able to spend time home with children and know their strengths and weaknesses in terms of studies and have family time with them.

It is important to note that the perceived positive benefits of working from home are class related with some respondents acknowledging their privilege. For others,

Some things are much better for our family. My partner is no longer commuting to work and is working from home. Our teenager is home-schooling and much happier. But we also are very privileged and have a large home and money.

Some people commented on the saving of time and money due to being able to work from home:

On the bright side, I feel like I save a lot of time and stress by not having to commute. I feel my life now is quieter than before the pandemic with less trips for meetings and conferences.

For others, the pandemic provided much-needed time for reflection where:

The pandemic had changed my professional life with a positive effect: I reassessed my place in profession as a practitioner.

## Expectations on the self

In this theme, we explore the notion of expectations that respondents placed on themselves while working from home and how some respondents were able to negotiate more sustainable levels of expectations. The conditions of working from home and learning during COVID-19 have influenced people’s personal and employer expectations. This has ranged from introducing work stress from the public level into the private level of the home, especially when the private homelife has also changed as exemplified by this respondent:

My older children returned home creating financial stress in the household also increasing my house duties including cooking and cleaning.

Another respondent articulated her experience as follows:

I am a mother, wife, daughter, sister, friend, colleague and fulltime academic. Remote emergency teaching and learning has had a tremendous impact on my life. I had [to] become a fulltime home-school teacher to my 7-year-old along with occupying my 2-year fulltime. Despite having my husband working from home, when my children want me, they want me. This was very stressful as the demands of academia are merciless.

Some respondents experienced that working from home during COVID-19 has eased personal expectations. One respondent has reported this as, ‘I am experiencing less pressure to be a high performer in academia, and this is a relief’. The cognitive self-regulation required to manage ideas around expectations was possible for some, where others were still pressured to continue to perform according to a work environment that was unrelentless. This reality is summarised below by one of the respondents as follows:

I feel like my workload has tripled. We are moving at the same/faster rate than prior to COVID-19. Since the university has protocols in place to protect students (more or less, that’s the focus), it has been the workforce that carried out the protocols. This includes moving courses (like community practice) online, which are hard to teach in the ways I teach my classes. We are ‘adaptive and resilient’ which is also code for working incredibly long hours (I have averaged 10–15 hours/day, including most weekends working 5 hours/day, since August). We had extra training throughout the summer (and on 9-month contracts), and while we are very family focused and thoughtful about childcare/caregiving, the workload has not lessened overall, so the burden of work falls on the shoulders of those without caregiving – I am all for childcare/caregiving, but the workload overall should be less under those circumstances. In addition, we are trying to decolonize our syllabi, work towards justice, adopt better anti-racist, anti-oppressive policies, and manage COVID-related issues – attendance, sick students, mental [ill] health, shifts in field [placements], eye strain. We are on computers all the time and doing damage to our bodies. We are not able to ‘be in community’ or ‘be in the community’ so there’s a sense of loss, a challenge in how we engage, and limited person-to-person connections. It is lonely. The rat race continues; people are doing studies like this one and submitting new IRBs and writing lots and lots, while I don’t know how to do all the writing/research with my current workload. It is all too much and feels like we are holding up a system that is going to crash down – it feels like a huge weight that keeps pressing on you. I have been depressed, cried at least once/week, considered leaving academia, been angry at the system, and been neglectful of other relationships.

## Coping with the challenges of working from home

The coping research literature generally posits that some strategies do not lead to better outcomes for people. Termed as avoidance strategies, participating individuals can see solutions in removing oneself from uncomfortable situations. Obviously, such strategies are not possible when people are on lockdown. This approach risks developing strategies that are unhealthy, such as excessive drinking or the constant engagement with the news as identified by one respondent:

I would say my wine intake has increased for sure, especially on the weekends, also watching news and doom scrolling in social media has negatively impacted my thought.

However, most respondents in this survey articulated coping strategies rather than avoidance strategies. As outlined above, a problem-orientated approach first focuses on identifying a problem and then seeks to resolve it. For instance, many respondents felt that isolation from peers was an issue. To address this, many respondents stated that they tried to stay in contact with colleagues and ‘maintain the relationship with friends, students, colleagues, community members and all other people’ whom they normally socialised with pre-COVID-19. One respondent volunteered on a COVID-19 psychosocial support hotline to have contact with other people while others used their resources on ‘virtual platforms to maintain connections with friends’. Some respondents used rational approaches to their work and made commitments to ‘escape from my job tasks’ and create quality time with family. Respondents used cognitive strategies to reformulate their understanding of their reality and engaged in new theories and philosophies that helped them understand their work and place in the world in a different way. For example, one respondent ‘discovered Eco-social work which is my new obsession’. Connecting with professional support, such as a psychologist, to help mediate the challenges of working from home was also common.

By far, the most common strategies respondents used to cope with the working from home were emotionally-orientated strategies. Many respondents referred to the idea of managing their thought process as a way of controlling their emotional reactions to their situations. For some, this included the idea of ‘self-acceptance in a pandemic situation as a reality that must be faced’, and it was important to ‘just tak[e] each day at a time’. For others, it was important to ‘avoid having conversations with people who are hysteric[al] [*sic*] about it [COVID-19]’. Respondents reported (re)experiencing nature during the time spent working and learning from home. It is important to highlight the class element in these responses, a point acknowledged by some of the respondents. For the majority of people under lockdown, a garden is not a reality nor is the ability to take oneself to a forest or beach. However, many respondents in this survey did have these resources and used them in a way to help manage their stress. One respondent claimed, ‘I have always enjoyed spending time outdoors, so I didn’t “reconnect” so much as I kept connecting’. Another enjoyed ‘taking photos of plants when on my daily walk. [This] allowed me to document the changes in seasons – marking time’. For others,

Gardening has become the focus of my daily existence. To plant or even to sit quietly is really therapeutic for me. It has become a big part of my mental health care management. To be outside in nature during the changing seasons is very good for my mood.

Religion was important to many of the respondents. Some commented that they deepened or discovered their faith during this period and turned to God to lead them and their country. ‘Here is a deep sense that this country needs God’s leading’, said one respondent. Others had connected with God in a special way. One respondent claimed, ‘I had more time to read my Bible, pray and gave fellowship with other believers in WhatsApp platforms’. The belief that each of us needs to use life as opportunities for learning was presented by another respondent who stated that they:

Embrac[ing] the situation and accepting that the pandemic is a test from God sent to all of mankind [*sic*]. In it, there is lesson for each [one] of us. I worked hard to search for my lesson, learn from it and grow as person. A greater consciousness of Allah (our Creator) has become my greatest coping strategy.

More commonly, coping strategies associated with spirituality were also reported. These included Qi Gong, meditation, yoga and trying to ‘live in the now’.

## Conclusion and implications of the research findings

This article set out to highlight social work communities’ experiences of working and learning from home. The results showed that generally respondents, of which women were over-represented, found working and learning from home challenging, but in the main, they deployed constructive and self-regulatory coping strategies. At the same time, this research highlights the emotionality of working and learning from home during COVID-19, for both teaching and studying social work. Respondents struggled with finding placements for students and feeling inadequate in their work performance. They also struggled with managing the private and public boundaries calling into question issues of the encroachment of the work life into the home. Given the vast majority of respondents identified as women (80%), we can deduce that there was also a gendered element to the responses with respondents describing the pressure of having many roles to manage within the space of the home. The lack of clarity as to when the pandemic will end, and when people can go back to teaching and studying as before loomed over the respondents’ answers. Student placements were difficult to arrange, and many students had to undertake placements online. What longer term implications this may have on student’s professional development has yet to be appreciated.

By understanding the experiences of social work educators and students, we can help management staff address some of the stressors involved in working and learning from home. It is also important to give voice to social work educators’ and students’ experiences so that connections can be made with those of other professionals whose reality is also one of working from home (Xiao et al., 2021). Furthermore, the use of remote technologies in delivering social work education and practice has changed working practices in social work communities so dramatically that these merit an examination of how individuals adjust (or not) to this new workspace.

Understanding these experiences will help us better understand the links between individual experiences, social positioning and social structures, particularly those linked to structural inequalities. Crucial among these is that of researching the conflation of workspace and home-space when both are conflated into one, particularly through a gender lens, given the majority of respondents were women and faced the implications of gendered roles in the home. The merging of different spheres previously located within the public (work) and private (home) spaces, and the daily routines carried out within each, removes a barrier to leaving workplace stresses in the office. Furthermore, the arrangements for self-care – an integral part of social work practice (Janika et al., 2020) – and the coping strategies that the social work community, educators and students utilised to get through these new work and study arrangements merit investigation.

Consequently, this research explored the issues arising from the merging of previously separated spheres and their different routines given that both are now conducted within the home-space. Furthermore, the coping strategies used by social work communities to implement these new work and study arrangements are of concern. Given that self-care is considered an integral part of social work practice, it has to be realised by educators and students alike.

This research contributes to a wider understanding of the experiences of working and studying from home during COVID-19, focusing specifically on social work perspectives. The findings are also relevant for administrators who are responsible for the welfare of their employees and students, and for educators in the management of their courses.

## Limitations

The direct voice of service users is missing from examining how they have coped with the mass move to working from home as an issue that has exercised other members of social work communities. Service users play an important role in social work education in many countries around the world. We are aware that the direct voice of service users is missing from our research, due mainly to the research sample self-selecting through the IASSW website. Thus, recruiting from a larger population is necessary to contribute to filling that gap in future. We are aware that the sample is not representative of all educators and students in the different social work communities and is skewed towards women social work educators due to the respondent’s self-selection. We are also aware that countries experienced various restrictions, so any generalisation of these results needs to consider the varied contexts within which responses occur.
